# The Hinge Region Strengthens the Nonspecific Interaction between Lac-Repressor and DNA: A Computer Simulation Study

**DOI:** 10.1371/journal.pone.0152002

**Published:** 2016-03-23

**Authors:** Lili Sun, Marcin Tabaka, Sen Hou, Lin Li, Krzysztof Burdzy, Aleksei Aksimentiev, Christopher Maffeo, Xuzhu Zhang, Robert Holyst

**Affiliations:** 1 Institute of Physical Chemistry PAS, Kasprzaka 44/52, 01–224, Warsaw, Poland; 2 Computational Biophysics and Bioinformatics, Department of Physics, Clemson University, Clemson, South Carolina, 29634, United States of America; 3 Department of Mathematics, University of Washington, Seattle, Washington, 98195–4350, United States of America; 4 Department of Physics, University of Illinois, Urbana, Illinois, 61801, United States of America; Weizmann Institute of Science, ISRAEL

## Abstract

LacI is commonly used as a model to study the protein-DNA interaction and gene regulation. The headpiece of the lac-repressor (LacI) protein is an ideal system for investigation of nonspecific binding of the whole LacI protein to DNA. The hinge region of the headpiece has been known to play a key role in the specific binding of LacI to DNA, whereas its role in nonspecific binding process has not been elucidated. Here, we report the results of explicit solvent molecular dynamics simulation and continuum electrostatic calculations suggesting that the hinge region strengthens the nonspecific interaction, accounting for up to 50% of the micro-dissociation free energy of LacI from DNA. Consequently, the rate of microscopic dissociation of LacI from DNA is reduced by 2~3 orders of magnitude in the absence of the hinge region. We find the hinge region makes an important contribution to the electrostatic energy, the salt dependence of electrostatic energy, and the number of salt ions excluded from binding of the LacI-DNA complex.

## Introduction

LacI, which controls gene expression of the proteins involved in lactose metabolism in enteric bacteria such as *Escherichia* coli, is commonly used as a model for DNA-binding proteins. LacI searches for its specific binding site among a huge number of nonspecific binding sites on DNA. Nonspecific binding is of paramount important because it can accelerate the searching process by facilitated diffusion (such as sliding, hopping along DNA) for the specific binding site. In the facilitated diffusion, the LacI binds nonspecifically to DNA [1–5]. The hinge region of the LacI has been reported to play a crucial role in the specific binding of the LacI and DNA [6]; however, the factors that influence the nonspecific binding process have not been elucidated. Several early reports implied that the hinge region could also play an important function in nonspecific binding. Furini *et al*. [7] used the headpiece structure without the hinge region to study the nonspecific sliding of LacI along the major groove of the DNA. They found the free energy barrier for sliding to be about ca. 14.68 kT and the corresponding diffusion coefficient to be approximately 2.5×10^2^ to 1×10^3^ bp^2^/s, close to the lower limit of the experimental data [8]. On the other hand, Marklund *et al*. [9] used the headpiece structure with the hinge region to study the same sliding process. They found the energy barrier for sliding to be about 1.0 kT (fifteen folds lower than Furini *et al*.'s estimate [7]) and the corresponding diffusion coefficient to be 1.23×10^6^ bp^2^/s [9], close to the upper limit of the experimental data [4,8]).Kalodimos *et al*. found that the hinge regionplays an important role in the transition from nonspecific to specific binding [10]. In the case of specific binding, the hinge region forms an α helix and embeds itself into the minor groove of the DNA fragment [10–11]. In the nonspecific binding case, the hinge region is disordered and does not make contact with the DNA. Therefore, a complete assessment of the contribution of the hinge region to the nonspecific interaction between the LacI and DNA is needed.

The LacI protein contains two DNA binding subunits. Each DNA binding subunit contains two monomers, each of which is composed of four distinct regions: the N-terminal DNA-binding domain (residues 1–46), the hinge domain (residues 47–62), the core domain (residues 63–340) and the C-terminal tetramerization domain (residues 341–357) [12–13]. The DNA-binding domain and the hinge region form the headpiece of LacI (residues 1–62), which we hereafter denote as LacIΔ1–62. Due to the absence of the structure of the full-length LacI nonspecifically bound to DNA, the NMR structure of LacIΔ1–62 has been commonly used for computer simulation studies of LacI's nonspecific binding [6–7,9–10,14]. Biochemical studies have shown the LacIΔ1–62 protein, which contains a disulfide bond connecting the two protein monomers, to have similar binding affinity to DNA as the whole LacI complex [6]. Because of this high binding affinity and its relatively small size, the LacIΔ1–62 structure has been widely used for the studies of nonspecific interactions between LacI and DNA [6–7,9–10,14–15].

In order to elucidate the contribution of the hinge region to the nonspecific binding of LacIΔ1–62 to DNA, we used the NAMD [16] and the umbrella sampling technique [17] to compute the dissociation free energy of LacI from DNA with and without hinge region. Continuum electrostatic calculations [18] elucidated the electrostatic interaction between the hinge region and DNA and its dependence on the salt concentration. We also analyzed the hydrogen bonds between the hinge region and DNA over the course of the molecular dynamics (MD) [19] trajectory.

## Results and Discussion

### The conformation of nonspecific LacIΔ1-62/DNA complex

**Fig 1:**
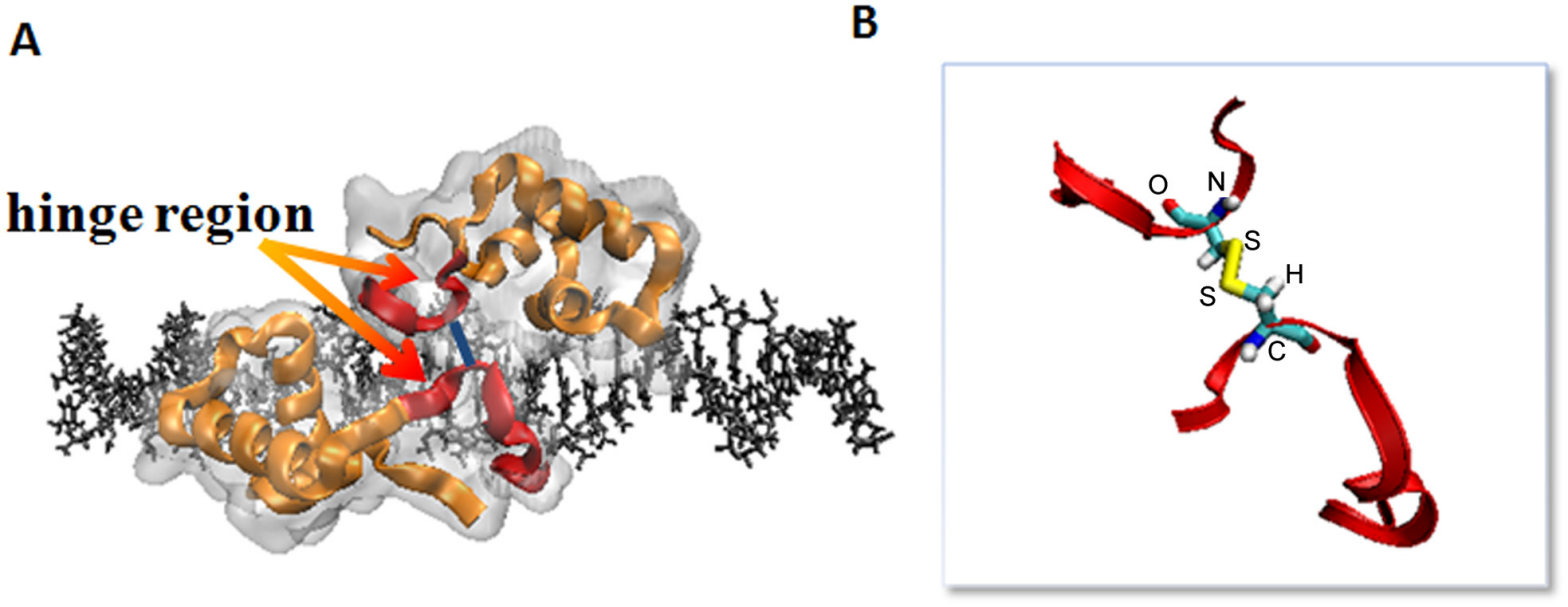
The conformation of the nonspecific LacIΔ1-62/DNA system. (A) The LacIΔ1–62 is composed of two monomers, which are connected by an S-S bond in the residue CYS52 (blue line). (B) explicitly represents the S-S bridge. The DNA-binding domain of LacIΔ1–49 is shown as orange ribbons; The hinge region of LacIΔ50–62 is shown as red ribbons; The residue CYS52 is shown in Licorice representation; S atoms are in yellow, O atoms are in red, N atoms are in blue, C atoms are in cyan, and H atoms are in white.

Fig 1A shows the structure of the nonspecific LacIΔ1-62/DNA. The headpiece is divided into a structured region (residues 1–49) and an unstructured region (residues 50–62) by Kalodimos *et al*. [6], who first provided the NMR structure of the nonspecific LacIΔ1-62/DNA complex. The structured region (residues 1–49) folds up into α helices and is deeply embedded in the major groove of the DNA and is directly in contact with the surface of the DNA. The unstructured region (residues 50–62) is disordered and connected by an S-S bond between two monomers (Fig 1B). The binding of the LacIΔ1–62 to the nonspecific sequence does not induce α helix formation in the hinge region. This unstructured region forms an α helix when engaged in specific binding with DNA, while keeping a free state in the nonspecific complexes [10]. The diameter of the DNA double helix is ca. 2 nm, and the LacIΔ1–62 is embedded ca. 1 nm in the major groove of the DNA (Appendix C in S1 File). In our study, we deleted the unstructured region to investigate the interaction energy between the DNA and the LacI with or without the hinge region. As the structured region is mainly the DNA binding domain and the unstructured region is mainly the hinge region, we call the structured region the DNA binding domain and the unstructured region the hinge region in this study.

### The contribution of the hinge region to the free energy for the micro-dissociation process

**Fig 2:**
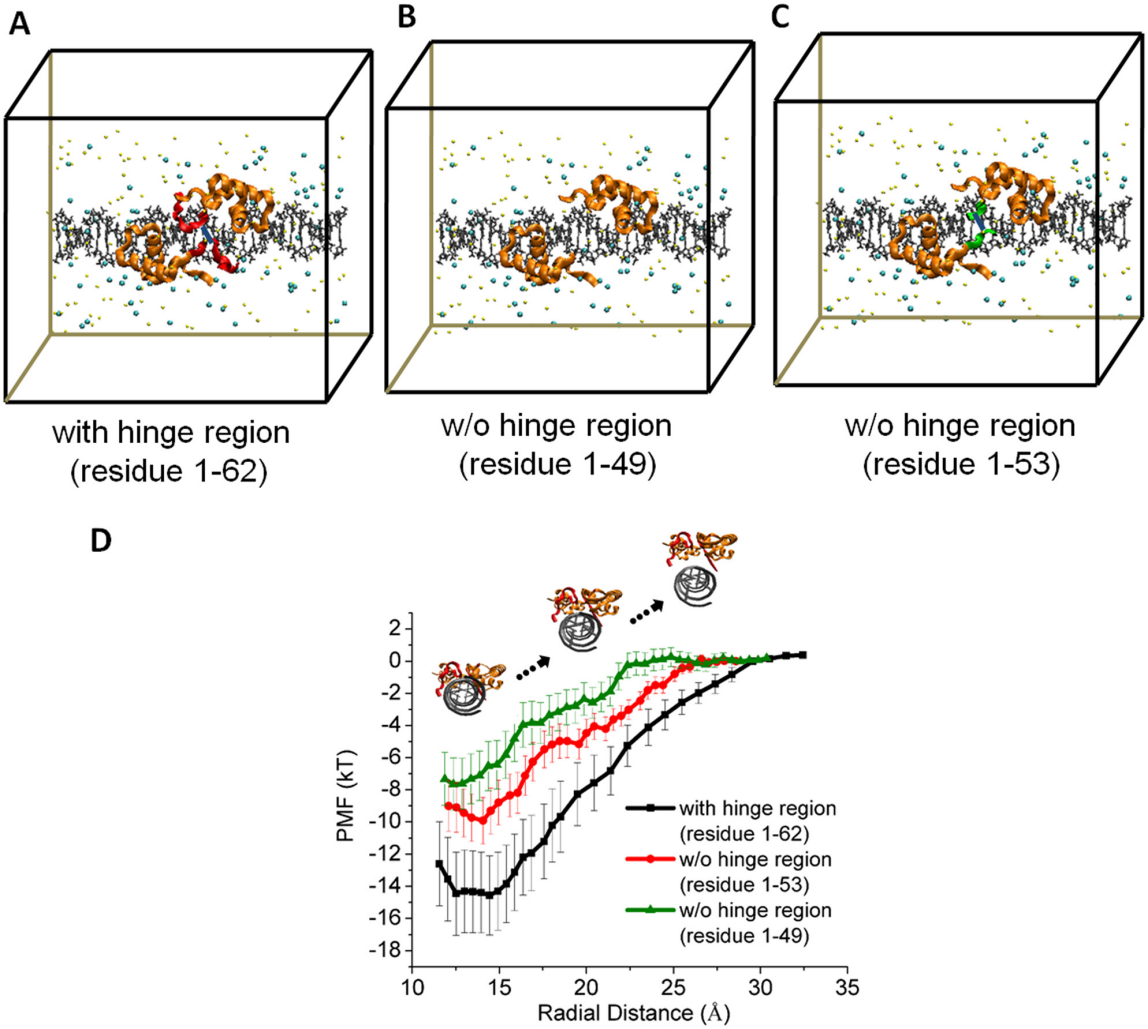
The micro-dissociation PMF of the LacI from nonspecific DNA. (A) PMF during the micro-dissociation process of the LacI from DNA both with the hinge region and without the hinge region. The PMF of LacIΔ1–62 micro-dissociation from DNA is shown by the black curve; The PMF of LacIΔ1–53 from DNA is shown by the red curve; The PMF of LacIΔ1–49 from DNA is shown by the green curve. (B) The initial simulation system containing the LacIΔ1–62, DNA and salt ions. Water molecules are not shown. The DNA (gray) is shown in licorice representation; residues 1–49 (orange) are shown in ribbons; residues 50–53 (green) are shown in ribbons; residues 54–62 (red) are shown in ribbons; sodium and chloride ions are shown as yellow and cyan spheres; (C) The initial simulation system containing the LacIΔ1–49, DNA and salt ions; (D) The initial simulation system containing the LacIΔ1–53, DNA and salt ions. The water box in the Fig 2 is schematic and does not reflect the real size of simulation box.

We calculated the free energy profile by using the structure of LacI with the hinge region and without the hinge region (LacIΔ1-62/DNA and LacIΔ1-49/DNA). Once the LacI has micro-dissociated from DNA, the LacI can rebind to the same DNA with some probability or it reaches a distance *R*_c_ to achieve macro-dissociation state, where rebinding is uncorrelated to the microscopic dissociation event [9]. We defined the micro-dissociation state as that the PMF curve gets flat because of the screening effect of the salt ions. The PMF value at micro-dissociation state is set as zero, since the constant value only parallel displaces the whole curve, but does not change its shape. The initial simulation system of LacIΔ1-62/DNA is shown in Fig 2A. We built a LacI without the hinge region (LacIΔ1-49/DNA) as a comparison system to show the contribution of the hinge region in the LacI (Fig 2B). The monomers in the LacI are connected by an S-S bond at residue 52 in the hinge region [6]. Simply deleting the hinge region will separate the two monomers from each other in MD simulation. Therefore, we kept the S-S bond between the two monomers and built a third simulation system of LacIΔ1-53/DNA (Fig 2C). We identified that both monomers dissociated away from the DNA simultaneously to roughly the same extent even if the two monomers were not connected by an S-S bond during the micro-dissociation process (Appendix D in S1 File). The free energy change for micro-dissociation can be calculated as:

$$\Delta \text{G}={\text{G}}_{\text{unbound}}-{\text{G}}_{\text{bound}}$$ (1)

where G_unbound_ is the free energy of the micro-dissociation state, which corresponds to the maximum in the potential of mean force or the free energy (PMF) curve (Fig 2D). G_bound_ represents the state where the LacI closely binds to the DNA, which corresponds to the minimum in the PMF curve (Fig 2D). With the hinge region, the LacIΔ1–62 needs ca. 14 kT to micro-dissociate from the DNA. LacIΔ1–53 needs ca. 9.5 kT to micro-dissociate from the DNA. LacIΔ1–49 needs ca. 7 kT to micro-dissociate from the DNA (Fig 2D). Without the hinge region, the free energy for micro-dissociation decreases by half. The hinge region not only affects the free energy for dissociation, but also the distance of micro-dissociation. We found that the radial distance of micro-dissociation decreased from 28 Å to 22 Å without the hinge region. Marklund *et al*. [9] used the Amber force field to calculate the PMF for dissociation of LacIΔ1-62/DNA system. They found that the LacI needed ca. 12 kT to dissociate from DNA at a radial distance of 28 Å [9]. Although we used a different force field for the MD simulation, we obtained a similar result (ca. 14 kT).

There is a minimum at ca. 13 Å for all the three PMF curves. At the minimal point, the LacI is not in as close contact with the DNA major groove as in the NMR's structure. Givaty *et al*. [20] also found that transcription factors diffuse along DNA in loose complexes, but not in close contact with the DNA major groove using coarse-grained models.

### The contribution of the hinge region to the electrostatic energy of the nonspecific LacIΔ1-62/DNA

Since the LacI is stabilized on nonspecific DNA by electrostatic interaction and a highly organized H-bond network [10,21], we explored the contribution of the hinge region to the electrostatic energy between the LacIΔ1–62 and DNA and also studied the hydrogen bond between the hinge region and DNA. Experimental data [10,22] show that salt ions decrease the association equilibrium constant K_obs_ between the LacI and DNA. This is, reflected in the formula

$$-\frac{\partial {\text{logK}}_{\text{obs}}}{\partial \text{log}\left[{\text{M}}^{+}\right]}=-\frac{\partial \Delta \text{G}}{\text{RTln}10\partial \text{log}\left[{\text{M}}^{+}\right]}$$ (2)

where [M^+^] is salt concentration, ΔG is the free energy for association, R is the gas constant, T is the temperature. At a fixed distance, the free energy depends only on the salt ion distribution [23]. Therefore, it is often assumed that ∂(Δ*G*_*el*_)/∂(*log*[M^+^]) roughly equals ∂(Δ*G*)/∂(*log*[M^+^]). The electrostatic energy is a linear function of the log [salt], the experimental slope is $\frac{\partial \left(\Delta G_{el}\right)}{\partial \left(log\left[{\text{M}}^{+}\right]\right)}$ = 26.79 kT/M.

**Fig 3:**
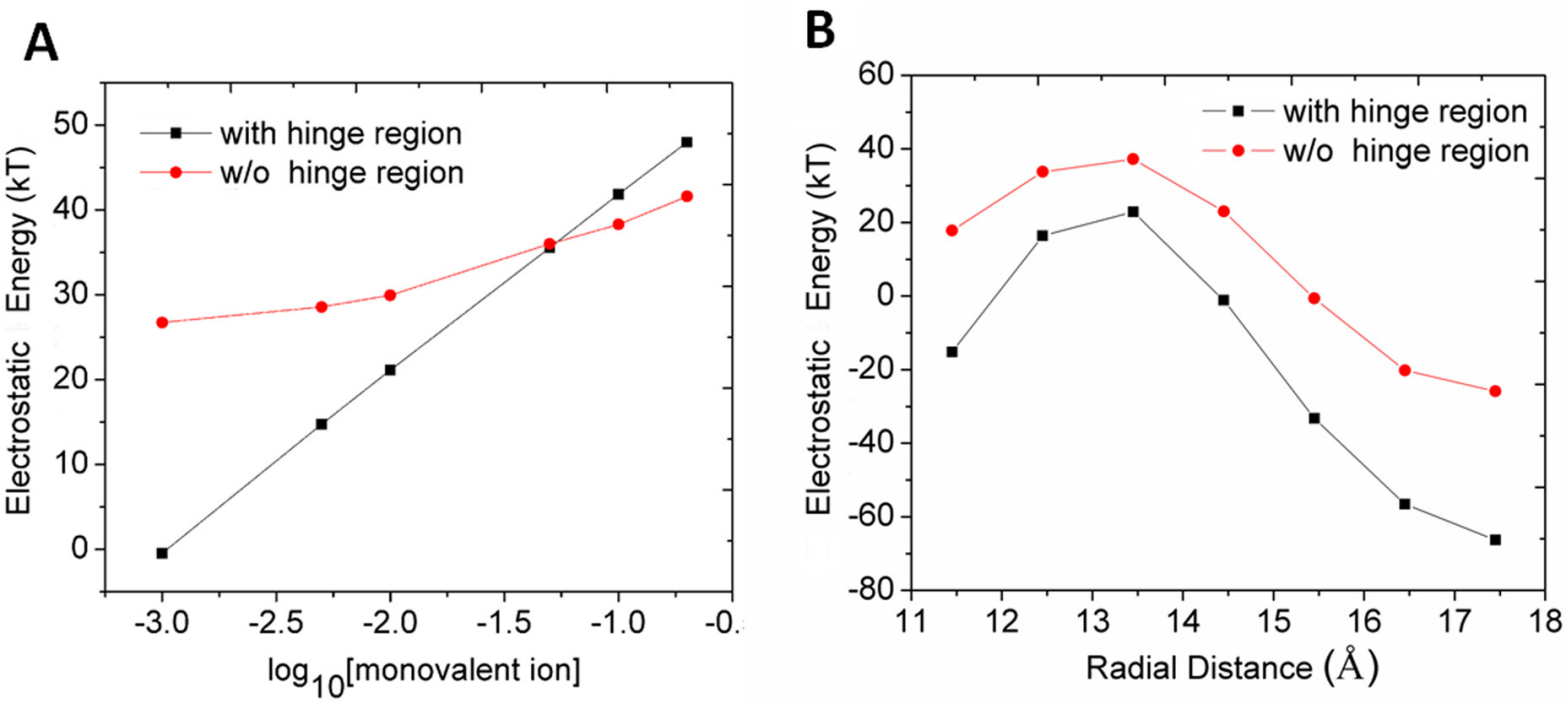
The electrostatic energy of nonspecific LacIΔ1-62/DNA and LacIΔ1-49/DNA. (A) The electrostatic energy as a function of salt concentration. (B) The electrostatic energy in pure water as a function of radial distance.

We also investigated salt dependence of hinge region on the free energy of the LacI/DNA. The radial distance between lacI and DNA is 11.45 Å, which corresponds to the closely binding LacI/DNA complex. The calculated ∂(Δ*G*_*el*_)/∂(*log*[M^+^]) for LacIΔ1-62/DNA was 21.51 ± 0.13 kT/M, which is roughly close to the experimental data. Without the hinge region, the ∂(Δ*G*_*el*_)/∂(*log*[M^+^]) for the LacIΔ1-49/DNA was 6.67 ± 0.76 kT/M (Fig 3A). Therefore, the hinge region enhances the salt dependence of the free energy of the LacI/DNA.

We also found that the counter ions released upon binding decreased without the hinge region. The number of counter ions released upon binding can be calculated from the slope of the salt dependence of electrostatic energy of the LacI with DNA according to Eqs S11-12 (Appendix B in S1 File). We found that 10.36 ions are released into solution when LacIΔ1–62 binds with DNA; and 3.29 ions are released into solution when LacIΔ1–49 binds with DNA. We also explored the ion atmosphere observed in MD simulations (Appendix F in S1 File). We calculated the charge of LacIΔ1–62 and DNA at neutral PH using pdb2pqr web server. The 18-base-pair nonspecific DNA fragment with protonation has -34 e negative net charges, the DNA binding domain has 0 e net charges, and the hinge region of the LacI shows +4 e positive net charges. The positive charges in the hinge region strongly interact with the negative charges of DNA. Therefore, the contribution of the hinge region to stabilize the LacI/DNA complexes is important and cannot be neglected.

We studied the electrostatic energy during the micro-dissociation of the LacI and DNA with and without the hinge region. The equations to calculate the electrostatic energy are shown in Eqs S1-10 (Appendix A in S1 File). Without the hinge region, the electrostatic interaction between the LacI/DNA is reduced, which is shown by an increase in the value of electrostatic energy in pure water (Fig 3B). There is a maximal point between 13 Å and 14 Å in Fig 3B. This maximal point is the counterbalance of the increased coulombic energy and the decreased solvation energy (Appendix G in S1 File).

### The hydrogen bonds between the hinge region and DNA in nonspecific LacIΔ1-62/DNA

**Fig 4:**
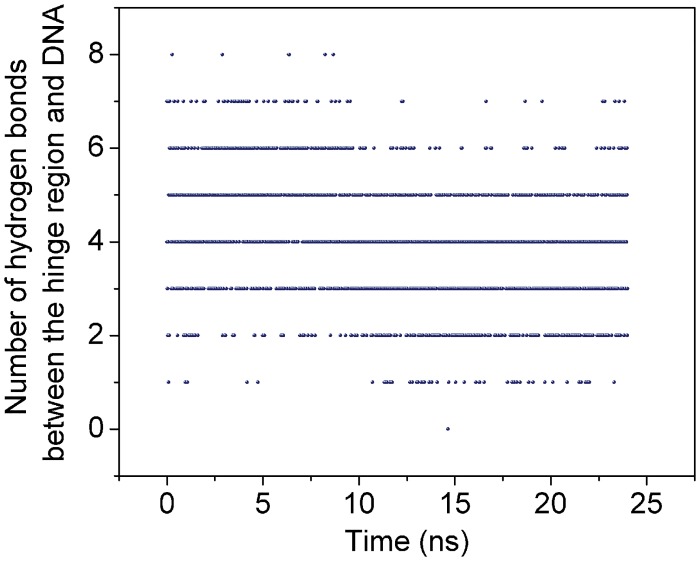
The number of hydrogen bond along the trajectory between the hinge region of the LacI and DNA.

We also studied the hydrogen bond between the hinge region and DNA with LacIΔ1-62/DNA system with unrestrained MD simulation after equilibration. The formation of a hydrogen bond is identified when the donor and acceptor atoms are closer together than 3.0 Å and the angle of donor-hydrogen-acceptor is between 150° and 180°. The average number of hydrogen bonds is 4±1.3. The possibility of hydrogen bond formation is larger than that with the number of hydrogen bonds as 3, 4 or 5 (Fig 4).

We also listed the top 3 occupancies for the hydrogen bonds in the hinge region and DNA. The highest occupancy ofthe hydrogen bond was between side N atom of ARG residue in the LacI and O atom of CYT in the DNA (Table 1). Hydrogen bonds between the DNA binding domain and DNA are stronger than those between the hinge region and DNA. Furini *et al*. report that 8 hydrogen bonds can be formed between one monomer of the DNA binding domain (residues 1–46) and DNA with occupancies larger than 50% [7], however, only 3 hydrogen bonds with occupancies larger than 50% are found between the two monomers of the hinge region and DNA. Therefore, the hydrogen bond between the hinge region and the DNA is not the main reason for the stabilization of the nonspecific LacI/DNA complex.

**Table 1:** H-bonds between the hinge region and DNA.

Donor	acceptor	H-bond occupancy^a^
PRO(A):ARG51:NH1(side)	DNA(D):ADE16:O4'(side)	59.28%
PRO(B):ARG51:NE(side)	DNA(C):CYT18:O1P(side)	83.28%
PRO(A):ALA53:N(main)	DNA(C):CYT18:O1P(side)	72%

^**a**^ The occupancy of the H-bonds is estimated as the percentage of the MD trajectory when donor and acceptor atoms are closer than 3.0 Å and the angle of donor-hydrogen-acceptor is between 150° and 180°

The LacI accelerates the search for its target on the DNA through facilitated diffusion [24] (e.g. one-dimensional sliding movement, hopping between adjacent binding positions, translocation between distant regions of the DNA). During the facilitated diffusion process, the LacI often micro-dissociates from the DNA to achieve an increased mobility. Based on the calculated free energy (Fig 2) during the micro-dissociation process of the LacI from the DNA, we discussed the influence of the hinge region on the micro-dissociation rate constant. The micro-dissociation rate constant is calculated through the integrals of the PMF curve in Fig 2. The equation for the micro-dissociation rate constant is as follows [9]:

$${\text{k}}_{\text{d}}^{\text{micro}}=\tau_{\text{d}}^{-1}=\int_{{\text{r}}_{0}}^{\rho }\frac{{\text{e}}^{-{\text{G}}_{\text{b}}\left(\text{r}\right)}}{{\text{D}}_{3}}\int_{{\text{r}}_{0}}^{\rho }{\text{e}}^{{\text{G}}_{\text{b}}\left({\text{r}}^{\prime }\right)}\int_{{\text{r}}_{0}}^{{\text{r}}^{\prime }}{\text{c}}_{0}\left({\text{r}}^{\prime \prime }\right){\text{dr}}^{\prime \prime }{\text{dr}}^{\prime }\text{dr}$$ (3)

where D_3_ = 50 μm^2^/s based on the Stokes-Einstein equation and we assumed that D_3_ does not change with or without the hinge region, τ_d_ is the microscopic residence time (the mean time to reach the endpoint), r_0_ corresponds to the LacI closely bound to the DNA, c_0_ is calculated as

$${\text{c}}_{0}\left(\text{r}\right)={\text{e}}^{-{\text{G}}_{\text{b}}\left(\text{r}\right)}/\int_{{\text{r}}_{0}}^{\rho }{\text{e}}^{{\text{G}}_{\text{b}}\left({\text{r}}^{\prime }\right)}\text{dr}$$ (4)

r_0_ ≤ r ≤ ρ, ρ is the endpoint, and also the radial distance of micro-dissociation. The microscopic dissociation rate increases by 3 orders of magnitude if we delete the hinge region (Table 2). The corresponding microscopic residence time decreases by 2~3 orders of magnitude without the hinge region. Herein the hinge region contributes to the micro-dissociation rate constant of the LacI from the DNA. Also by a comparison of the LacIΔ1-49/DNA to LacIΔ1-53/DNA, we report that the linkage between two monomers by an S-S bond increases the microscopic residence time ca. 7 times. This observation agrees with Kalodimos *et al*.'s study [6] showing the formation of a dimer is essential for the nonspecific binding of the LacI to DNA. Our result suggests that the hinge region stabilizes nonspecific LacIΔ1-62/DNA complex.

**Table 2:** The micro-dissociation rate constant of the LacI from the DNA.

LacI/DNA	The microscopic dissociation rate (1/s)
LacIΔ1-49/DNA	1.00 × 10^6^
LacIΔ1-53/DNA	1.53 × 10^5^
LacIΔ1-62/DNA	1.75 × 10^3^

The PMF curve is different from the curve of electrostatic energy during the micro-dissociation process. Especially, at a radial distance of 13 Å to 14 Å, the PMF curve shows a minimum value (Fig 2), while the electrostatic energy curve shows a maximum value (Fig 3A and Appendix K in S1 File). The electrostatic energy is part of but not equal to the free energy. The non-electrostatic interaction (for example, van der Waals' interaction and entropy) also contributes to the difference between the two. The calculation methods for the two energies are slightly different in their model and assumption. For example, in the calculation of electrostatic energy, water is treated as a simple dielectric medium and the effect of the relative motion and vibration of atoms is not considered; whereas in the calculation of free energy, the water molecules are represented explicitly in all-atom MD simulations and the relative motion of atoms is included. Nevertheless, both calculation methods are reliable and provide sufficient accurate description of the system [9,22]. In this study, we keep the same conformation during the micro-dissociation process to keep the rigidity of the structure; meanwhile the specific effect of the hinge region is reflected by comparing the difference of electrostatic energy with or without hinge region. Summarizing: The electrostatic energy is merely part of the free energy and its value differs significantly from the free energy. Therefore, substituting the free energy by the electrostatic energy might lead to a considerable qualitative and quantitative error when determining the equilibrium configuration of the system, which should be calculated by free energy (the PMF).

We note that the finite duration of our simulations implies that some slow degrees of freedom will not have been fully sampled and the corresponding entropy is not reflected in our PMFs. In particular, the LacI protein did not explore the complete orientational space available in the umbrella sampling simulations in the micro-dissociation state. However, since the orientation was explored to quite similar extents for the three systems, the PMFs should be offset in the micro-dissociation region by the same constant. Similarly, the hinge is disordered with a very large configurational space that the umbrella sampling simulations have not sampled completely. However, the configurational entropic free energy due to the hinge region that the simulations could not sample can be expected to provide a nearly uniform offset since the hinge is unfolded not only in the micro-dissociation state, but also in the nonspecifically bound state.

## Conclusion

In conclusion, we found that the hinge region plays an important role in the nonspecific binding of LacI to DNA. The hinge region forms an α helix and is embedded into the minor groove of DNA when the LacI binds specifically to DNA [10]. However, for nonspecific binding, although the hinge region keeps disordered and does not form an α helix, we found the hinge region contributes 50% to the stabilization of the LacIΔ1-62/DNA. Without the hinge region, the free energy for micro-dissociation of the LacI from DNA decreases from 14 kT to 7 kT, thus the microscopic dissociation rate increases ca. three orders of magnitude. The hinge region stabilized the LacIΔ1-62/DNA complex mainly through electrostatic interaction among protein, DNA and salt ions. Without the hinge region, the binding electrostatic energy increased by 23 kT and the number of salt ions excluded upon binding decreased from 10.36 to 3.29. Therefore, the hinge region should not be neglected in some cases, for example, if the micro-dissociation process is simulated. In our study, multiple computer simulation methods were used to show the broad influence of the hinge region on the nonspecific interaction. Our study helps to recognize the important role of the hinge region in stabilizing the nonspecific LacIΔ1-62/DNA complex.

## Methods

### Molecular models in the molecular dynamics simulation

The structure of the nonspecific LacIΔ1-62/DNA complex was taken from the PDB database (PDB ID is 1OSL) [10]. In this structure, the LacIΔ1–62 is composed of two monomers, which are connected via a disulfide bond of residue 52 in the hinge region. In this study, all the calculations on the LacIΔ1–62 refer to those of the dimer. The DNA fragment was extended from 18 to 30 nucleotides. The structure of the 12 nucleotides missing in the NMR data was added using the online tool 3D-DART [25]. The nucleotide sequences of DNA were TTATCGCGATAAGATATCTTATCGCGATAA. In order to avoid the end-to-end aggregation of DNA fragments, an ester bond was defined between nucleotide 1 and nucleotide 30 of each strand in order to simulate the DNA molecule with periodic boundary conditions [26]. The system was solvated by water molecules of 68 Å × 88 Å × 99 Å volume. The molecular systems of LacIΔ1-49/DNA and LacIΔ1-53/DNA were built based on the molecular system of LacIΔ1-62/DNA. The residues 50–62 and residues 54–62 were deleted from LacIΔ1-62/DNA, respectively. The structure information file was generated using psfgen [27]. Protonation at neutral pH states was used for all of the amino acids. The N terminal and C terminal were acetylated and amidated, respectively. Counter ions (Na^+^) were added to neutralize the system. These three systems were solvated in rectangular water box of 68 Å × 88 Å × 99 Å. Salt ions (Na^+^ and Cl^-^) were added until the salt concentration reached 0.2 M.

### Molecular dynamics method

All the atomistic MD simulations were performed using NAMD 2.9 [16] and the CHARMM-36 all-atom force field with CMAP correction [28]. The TIP3P model and the SETTLE algorithm [29] were used for water molecules [30]. The time step was set to 2 fs and the coordinates were saved every 9.6 ps. Long-range electrostatic interactions were treated by the Particle Mesh Ewald (PME) algorithm with a grid density of about 1.2 Å per grid point [31], and a 8 Å cutoff was used for short-range (non-bonded) interactions. The energy was first minimized by 2400 steps using the conjugate gradient method, followed by 2.19 ns NPT equilibration simulation. We proceeded with NVT production simulation after NPT equilibration simulation to keep the volume and density of the simulation system constant in all windows during umbrella sampling simulation. Langevin dynamics controlled the temperature at 298 K, using a strong damping factor (5.0 ps^-1^) during the NPT equilibration simulation and a weak damping factor (1.0 ps^-1^) during the NVT production simulations. Langevin pressure control was used to maintain a pressure of 1 bar, the piston period was 200 fs, and Langevin Piston Decay was 100 fs during the NPT equilibration simulation [32–33]. 24 ns of unrestrained MD simulation was performed to analyze the hydrogen bond between the hinge region and the DNA in the nonspecific binding.

We computed the potential of mean force (PMF) for the micro-dissociation [9] of the LacI with or without the hinge region from DNA using restrained MD simulation (the umbrella sampling technique) [17] and the weighted histogram analysis method (WHAM). The restrained MD simulation was performed on the backbone of the LacI and the DNA at a separating distance *r* during the micro-dissociation process of the LacI and the DNA. Every window was in a 0.5-Å interval of separating distance. We applied a harmonic spring of 10 kcal·mol^-1^·Å^-2^ to control the distance of the mass center between the backbone atoms of the LacI and the backbone atoms of the DNA. For the LacIΔ1-62/DNA system, the separating distance was in the range of 11 Å <*r*< 33 Å; For the LacIΔ1-53/DNA system, the separating distance was in the range of 12 Å <*r*< 29 Å; For the LacIΔ1-49/DNA system, the separating distance was in the range of 12 Å <*r*< 31 Å. Harmonic restraints are applied on the backbone atoms of LacI and DNA to force the distance between the mass centers of LacI and DNA to be close to the target distance. We rotated the LacIΔ1-62/DNA complex (PDB ID is 1OSL) to align the axis of DNA with the Z axis (see the structure of the LacIΔ1-62/DNA complex after rotation Appendix C in S1 File). The initial conformations for every window were generated iteratively by pulling the protein away from the DNA in the radial direction of the DNA axis (achieved by shifting the coordinate of protein atoms in the positive direction of the Y axis manually), which was followed by minimization and 2.19-ns NPT equilibration simulation for every window. The temperature, volume, total energy and number of water molecules between LacI and DNA in the first and the last umbrella sampling window of LacIΔ1-62/DNA system are shown in Appendix H in S1 File during NPT simulation. Restrained MD simulation was run 7.68 ns for every window. The radial distance between the LacIand the DNA was saved every 1000 time steps. The PMF was extracted using WHAM after restrained MD simulation [34]. We did another 7.68-ns restrained MD simulation for every window to calculate the PMF curves to prove the reproducibility of PMF curve (Appendix I in S1 File). We have identified that sufficient overlap between windows has been achieved for the LacIΔ1-62/DNA and LacIΔ1-49/DNA simulation system (Appendix J in S1 File).

### DelPhi calculation parameters and visualization of the molecular structure

The Y coordinates of LacI is shifted by Swiss-PdbViewer V3.7 software [35–36]. Missing hydrogen atoms, in all complexes and free molecules, were fixed by using the pdbxyz.x and the xyzpdb.x modules of TINKER software [37] with Amber99 force field parameters [38].

We investigated the electrostatic energy using DelPhi software at the following salt concentrations: *I* = 0 M, 0.001 M, 0.005 M, 0.01 M, 0.05 M, 0.1 M, 0.2 M. The biomolecules and the surrounding solvent were mapped onto a lattice, in which the grid spacing was 0.5 Å/grids. We took the geometric center of the LacI as the lattice center. The percentage of lattice filled was 70%. The dielectric constants of the biomolecules and solvents were set as 4.0 and 80.0, respectively. The solvent probe radius was 1.4 Å. The dipolar boundary condition was used. The force field parameters for radii and partial charges were taken from the Amber force field [38]. The potential root mean square change (RMSC) threshold was 0.0001 kT/e.

## Supporting Information

S1 File Supporting information with additional results and data. (DOCX)

## References

[1] Riggs AD, Bourgeoi S, Cohn M. Lac repressor-operator interaction.3. kinetic studies. J Mol Biol. 1970;53: 401–417. 4924006 10.1016/0022-2836(70)90074-4

[2] Berg OG, Winter RB, Vonhippel PH. Diffusion-driven mechanisms of protein translocation on nucleic-acids.1. models and theory. Biochemistry. 1981;20: 6929–6948. 7317363 10.1021/bi00527a028

[3] Gowers DM, Halford SE. Protein motion from non-specific to specific DNA by three-dimensional routes aided by supercoiling. Embo J. 2003;22: 1410–1418. 12628933 10.1093/emboj/cdg125 PMC151056

[4] Blainey PC, Luo GB, Kou SC, Mangel WF, Verdine GL, Bagchi B, et al Nonspecifically bound proteins spin while diffusing along DNA. Nat Struct Mol Biol. 2009;16: 1224–1229. 10.1038/nsmb.1716 19898474 PMC2889498

[5] Blainey PC, van Oijent AM, Banerjee A, Verdine GL, Xie XS. A base-excision DNA-repair protein finds intrahelical lesion bases by fast sliding in contact with DNA. P Natl Acad Sci USA. 2006;103: 5752–5757. 10.1073/pnas.0509723103 PMC1458645 16585517

[6] Kalodimos CG, Folkers GE, Boelens R, Kaptein R. Strong DNA binding by covalently linked dimeric Lac headpiece: Evidence for the crucial role of the hinge helices. P Natl Acad Sci USA. 2001;98: 6039–6044. 10.1073/pnas.101129898 PMC33418 11353825

[7] Furini S, Domene C, Cavalcanti S. Insights into the sliding movement of the lac repressor nonspecifically bound to DNA. J Phys Chem B. 2010;114: 2238–2245. 10.1021/jp906504m 20095570

[8] Wang YM, Austin RH, Cox EC. Single molecule measurements of repressor protein 1D diffusion on DNA. Phys Rev Lett. 2006;97: 048302 16907618 10.1103/PhysRevLett.97.048302

[9] Marklund EG, Mahmutovic A, Berg OG, Hammar P, van der Spoel D, Fange D, et al Transcription-factor binding and sliding on DNA studied using micro- and macroscopic models. P Natl Acad Sci USA. 2013;110: 19796–19801. 10.1073/pnas.1307905110 PMC3856812 24222688

[10] Kalodimos CG, Biris N, Bonvin AMJJ, Levandoski MM, Guennuegues M, Boelens R, et al Structure and flexibility adaptation in nonspecific and specific protein-DNA complexes. Science. 2004;305: 386–389. 15256668 10.1126/science.1097064

[11] von Hippel PH. Completing the view of transcriptional regulation. Science. 2004;305: 350–352. 15256661 10.1126/science.1101270

[12] Lewis M. The lac repressor. C R Biol. 2005;328: 521–548. 15950160 10.1016/j.crvi.2005.04.004

[13] Swint-Kruse L, Matthews KS. Allostery in the Lacl/GaIR family: variations on a theme. Curr Opin Microbiol. 2009;12: 129–137. 10.1016/j.mib.2009.01.009 19269243 PMC2688824

[14] Furini S, Barbini P, Domene C. DNA-recognition process described by MD simulations of the lactose repressor protein on a specific and a non-specific DNA sequence. Nucleic Acids Res. 2013;41: 3963–3972. 10.1093/nar/gkt099 23430151 PMC3627591

[15] Spronk CAEM, Bonvin AMJJ, Radha PK, Melacini G, Boelens R, Kaptein R. The solution structure of Lac repressor headpiece 62 complexed to a symmetrical lac operator. Struct Fold Des. 1999;7: 1483–1492. 10.1016/s0969-2126(00)88339-2 10647179

[16] Phillips JC, Braun R, Wang W, Gumbart J, Tajkhorshid E, Villa E, et al Scalable molecular dynamics with NAMD. J Comput Chem. 2005;26: 1781–1802. 16222654 10.1002/jcc.20289 PMC2486339

[17] Torrie GM, Valleau JP. Monte-carlo free-energy estimates using non-boltzmann sampling-application to subcritical lennard-Jones fluid. Chem Phys Lett. 1974;28: 578–581.

[18] Li L, Li CA, Sarkar S, Zhang J, Witham S, Zhang Z, et al DelPhi: a comprehensive suite for DelPhi software and associated resources. BMC Biophys. 2012;5:10.1186/2046-1682-5-9 PMC3463482 22583952

[19] Karplus M, Mccammon JA. Dynamics of proteins—elements and function. Annu Rev Biochem. 1983;52: 263–300. 6351724 10.1146/annurev.bi.52.070183.001403

[20] Givaty O, Levy Y. Protein sliding along DNA: dynamics and structural characterization. J Mol Biol. 2009;385: 1087–1097. 10.1016/j.jmb.2008.11.016 19059266

[21] Pabo CO, Sauer RT. Transcription factors-structural families and principles of DNA recognition. Annu Rev Biochem. 1992;61: 1053–1095. 1497306 10.1146/annurev.bi.61.070192.005201

[22] Misra VK, Hecht JL, Sharp KA, Friedman RA, Honig B. Salt effects on protein-DNA interactions-the lambda-ci repressor and ecori endonuclease. J Mol Biol. 1994;238: 264–280. 8158653 10.1006/jmbi.1994.1286

[23] Dahirel V, Paillusson F, Jardat M, Barbi M, Victor JM. Nonspecific DNA-protein interaction: why proteins can diffuse along DNA. Phys Rev Lett. 2009;102:10.1103/PhysRevLett.102.228101 19658903

[24] Tabaka M, Kalwarczyk T, Holyst R. Quantitative influence of macromolecular crowding on gene regulation kinetics. Nucleic Acids Res. 2014;42: 727–738. 10.1093/nar/gkt907 24121687 PMC3902910

[25] van Dijk M, Bonvin AMJJ. 3D-DART: a DNA structure modelling server. Nucleic Acids Res. 2009;37: W235–W239. 10.1093/nar/gkp287 19417072 PMC2703913

[26] Maffeo C, Luan BQ, Aksimentiev A. End-to-end attraction of duplex DNA. Nucleic Acids Res. 2012;40: 3812–3821. 10.1093/nar/gkr1220 22241779 PMC3351176

[27] Kale L, Skeel R, Bhandarkar M, Brunner R, Gursoy A, Krawetz N, et al NAMD2: Greater scalability for parallel molecular dynamics. J Comput Phys. 1999;151: 283–312.

[28] MacKerell AD, Bashford D, Bellott M, Dunbrack RL, Evanseck JD, Field MJ, et al All-atom empirical potential for molecular modeling and dynamics studies of proteins. J Phys Chem B. 1998;102: 3586–3616. 10.1021/jp973084f 24889800

[29] Miyamoto S, Kollman PA. Settle-an analytical version of the shake and rattle algorithm for rigid water models. J Comput Chem. 1992;13: 952–962.

[30] Jorgensen WL, Chandrasekhar J, Madura JD, Impey RW, Klein ML. Comparison of simple potential functions for simulating liquid water. J Chem Phys. 1983;79: 926–935.

[31] Essmann U, Perera L, Berkowitz ML, Darden T, Lee H, Pedersen LG. A smooth particle mesh ewald method. J Chem Phys. 1995;103: 8577–8593.

[32] Feller SE, Zhang YH, Pastor RW, Brooks BR. Constant-pressure molecular-dynamics simulation-the langevin piston method. J Chem Phys. 1995;103: 4613–4621.

[33] Martyna GJ, Tobias DJ, Klein ML. Constant-pressure molecular-dynamics algorithms. J Chem Phys. 1994;101: 4177–4189.

[34] Kumar S, Bouzida D, Swendsen RH, Kollman PA, Rosenberg JM. The weighted histogram analysis method for free-energy calculations on biomolecules.1. The method. J Comput Chem. 1992;13: 1011–1021.

[35] Guex N, Peitsch MC. SWISS-MODEL and the Swiss-PdbViewer: an environment for comparative protein modeling. Electrophoresis. 1997;18: 2714–2723. 9504803 10.1002/elps.1150181505

[36] Liu YZ, Wu M, Feng XZ, Shao XG, Cai WS. Adsorption behavior of hydrophobin proteins on polydimethylsiloxane substrates. J Phys Chem B. 2012;116: 12227–12234. 10.1021/jp304796p 22992191

[37] Ren P, Ponder JW. Tinker polarizable atomic multipole force field for proteins. Abstracts of Papers of the American Chemical Society. 2002;224: U473–U473.

[38] Weiner SJ, Kollman PA, Case DA, Singh UC, Ghio C, Alagona G, et al A new force-field for molecular mechanical simulation of nucleic-acids and proteins. J Am Chem Soc. 1984;106: 765–784.

