# Supplementary material for: The Hinge Region Strengthens the Nonspecific Interaction between Lac-Repressor and DNA: A Computer Simulation Study
Source: PLoS One. 2016 Mar 23;11(3):e0152002. doi: 10.1371/journal.pone.0152002 (PMC4805274; doi:10.1371/journal.pone.0152002)
Supplement: S1 File — (DOCX) [file pone.0152002.s001.docx]

**Supporting Information**

**The hinge region strengthens the nonspecific interaction between lac-repressor and DNA: a computer simulation study**

Lili Sun^1^, Marcin Tabaka^1^, Sen Hou^1^, Lin Li^2^, Krzysztof Burdzy^3^, Aleksei Aksimentiev^4^, Christopher Maffeo^4^, Xuzhu Zhang^1^ and Robert Holyst^1,*^

^1^Institute of Physical Chemistry PAS, Kasprzaka 44/52, 01-224 Warsaw, Poland

^2^Computational Biophysics and Bioinformatics, Department of Physics,Clemson University, Clemson, SC 29634, USA

^3^[Department of Mathematics](http://www.math.washington.edu/Temporary/NewDesign/index.php), [University of Washington,](http://www.washington.edu) [Seattle](http://www.seattle.gov/), WA 98195-4350, USA

^4^ Department of Physics, University of Illinois, Urbana, Illinois 61801, United States

^*^To whom correspondence should be addressed: Institute of Physical Chemistry PAS, Kasprzaka 44/52, 01-224 Warsaw, Poland Corresponding author: E-mail: [rholyst@ichf.edu.pl](mailto:rholyst@ichf.edu.pl)

**Table of Contents**

**Appendix A The calculation of electrostatic energy**

**Appendix B The calculation of ion exclusion from DNA when LacI binds to DNA**

**Appendix C The ensemble of LacI∆1-62/DNA complex**

**Appendix D The radial distance between two monomers of the LacI and DNA during the micro-dissociation process**

**Appendix E** **Salt dependence of the electrostatic energy of LacI/DNA with or without hinge region for 20 conformers**

**Appendix F Characterization of the ion atmosphere of LacI/DNA based on the results of the MD simulations**

**Appendix G Analysis of electrostatic energy barrier**

**Appendix H The temperature, volume, total energy and number of water molecules in the umbrella sampling windows**

**Appendix I The proof for the reproducibility of PMF curve for the LacI∆1-62/DNA and LacI∆1-49/DNA simulation system**

**Appendix J The window overlap for the LacI∆1-62/DNA and LacI∆1-49/DNA simulation system**

**Appendix K The electrostatic energy of nonspecific LacIΔ1-62/DNA and LacIΔ1-49/DNA as a function of radial distance**

**Appendix A The calculation of electrostatic energy**

Electrostatic energies were calculated with the finite difference Non-Linear Poisson Boltzmann (NLPB) Equation implemented in the Delphi software. Formation of complex AB by A and B at salt concentration I is

| $A(I)+B(I)\to AB(I)$ | (1) |
| --- | --- |

The change of electrostatic energy for binding $\Delta\Delta G_{\mathrm{el}}(I)$ in this process can be divided into three items: the change of coulombic interaction energy for binding $\Delta\Delta G_{\mathrm{coul}}\left( I \right)$, the change of the reaction field energy for binding $\Delta\Delta G_{\mathrm{react}}(I)$, and the change of the ion energy for binding $\Delta\Delta G_{\mathrm{grid}}(I)$.

| $\Delta\Delta G_{\mathrm{el}}\left( I \right)=\Delta\Delta G_{\mathrm{coul}}(I)+\Delta\Delta G_{\mathrm{react}}(I)+\Delta\Delta G_{\mathrm{gri}d}(I)$ | (2) |
| --- | --- |

The change of coulombic interaction energy for binding $(\Delta\Delta G_{\mathrm{coul}}\left( I \right))$ is

| ${\Delta\Delta G_{\mathrm{coul}}\left( I \right)=\Delta G}_{\mathrm{coul}}^{\mathrm{AB}}(I)-{\Delta G}_{\mathrm{coul}}^{A}(I) -{\Delta G}_{\mathrm{coul}}^{B}(I)$ | (3) |
| --- | --- |

The coulombic energy for AB, A or B is calculated using Eq. S4

| ${\Delta G}_{\mathrm{coul}}=\frac{1}{2}\sum_{j} q_{j}\varphi_{\mathrm{coul}}\left( \vec{r_{j}} \right)$ | (4) |
| --- | --- |

where $q_{j}$ is the charge of atom at $\vec{r_{j}}$. The coulombic potential $\varphi_{\mathrm{coul}}\left( \vec{r_{j}} \right)$ is generated by all the charges, of any kind, except for the one located at $\vec{r_{j}}$

| $\varphi_{\mathrm{coul}}\left( \vec{r_{j}} \right)=\sum_{i\neq j} \frac{q_{i}}{4\pi\varepsilon_{0}\varepsilon_{i}\left\vert\vec{r_{i}}-\vec{r_{j}} \right\vert}$ | (5) |
| --- | --- |

where $\left| \vec{r_{i}}-\vec{r_{j}} \right|$ is the vector difference of distance between charge i and charge j, $\varepsilon_{0}$ is the dielectric constant in vacumm, $\varepsilon_{i}$ is dielectric constant in the region where the charge of molecules are located, also called internal dielectric constant (INDI).$\varepsilon_{i}$is 4 in this paper. In order to investigate the effect of INDI, we also calculated the cases of$\varepsilon_{i}$=2, 3, 4 in supplementary data. (Fig S5)

The change of reaction field energy for binding ($\Delta\Delta G_{\mathrm{react}}(I)$) is denoted as

| $\Delta\Delta G_{\mathrm{react}}\left( I \right)={\Delta G}_{\mathrm{react}}^{\mathrm{AB}}(I)-{\Delta G}_{\mathrm{react}}^{A}(I) -{\Delta G}_{\mathrm{react}}^{B}(I)$ | (6) |
| --- | --- |

The corrected reaction field energy for AB, A or B is calculated through the Eq. S7

| ${\Delta G}_{\mathrm{react}}=\frac{1}{2}\sum_{j} q_{j}\varphi_{\mathrm{react}}\left( \vec{r_{j}} \right)$ | (7) |
| --- | --- |

where the corrected reaction field potential $\varphi_{\mathrm{react}}\left( \vec{r_{j}} \right)$ is generated by all the charges, of any kind, except for the one located at $\vec{r_{j}}$.

The corrected reaction field potential can be denoted in Eq. S8

| $\varphi_{\mathrm{react}}\left( \vec{r_{j}} \right)=\sum_{p} \frac{\delta_{p}}{4\pi\varepsilon_{0}\left\vert\vec{r_{i}}-\vec{r_{j}} \right\vert}$ | (8) |
| --- | --- |

where the polarization charge $\delta_{p}$ is computed from the induced charge. The induced charge at boundary point (l, m, n) is obtained from the numerical implementation of the Gauss law. These induced charges are then repositioned on the true molecular surface at a location defined by the intersection of the normal vector originating at the grid point and molecular surface [1].

The change of ion energy $\Delta\Delta G_{\mathrm{grid}}(I)$ describes the difference of total grid energy with and without salt.

| $\Delta\Delta G_{\mathrm{grid}}\left( I \right)=\left( \Delta G_{\mathrm{grid}}^{\mathrm{AB}}\left( I \right)-\Delta G_{\mathrm{grid}}^{\mathrm{AB}}\left( I=0 \right) \right)-\left( \Delta G_{\mathrm{grid}}^{A}\left( I \right)-\Delta G_{\mathrm{grid}}^{A}\left( I=0 \right) \right)-\left( \Delta G_{\mathrm{grid}}^{B}\left( I \right)-\Delta G_{\mathrm{grid}}^{B}\left( I=0 \right) \right)$ | (10) |
| --- | --- |

**Appendix B The calculation of ion exclusion from DNA when LacI binds to DNA**

We consider interaction of a charged ligand with a nucleic acid, in which the association involves the formation of $m^{'}$counterion pairs condensation and screening [2-4]. Once the complex forms, a number of $m^{'}$ions are released. If anion binding to the ligand is neglected and the solution is sufficiently dilute in all solute components, then $m^{'}$ can be calculated through [3-4]

| $-\frac{\partial logK_{\mathrm{obs}}}{\partial log[M^{+}]}=m^{'}\varphi$ | (11) |
| --- | --- |

The ion concentration is only correlated with the electrostatic energy, thus

| $-\frac{\partial logK_{\mathrm{obs}}}{\partial log[M^{+}]}=-\frac{\partial\Delta G_{\mathrm{el}}^{\theta}}{\mathrm{RTln}10\partial log[M^{+}]}=m^{'}\varphi$ | (12) |
| --- | --- |

Where $\Delta G_{\mathrm{el}}^{\theta}$ is the standard electrostatic interaction energy, $K_{\mathrm{obs}}$ is the association constant of LacI/DNA complex formation. $\varphi$ is expressed as the fraction of a counterions bound at the thermodynamic equilibrium, and is known for the particular DNA involved in the interaction (for native DNA, $\varphi$ is 0.88) [3].

**Appendix C The ensemble of LacI∆1-62/DNA complex**

In MD simulation, all the work based on the nonspecific LacI**∆**1-62/DNA complex originates from the research of Kalodimos *et al*. [5] (1OSL). The authors obtained at least 400 conformers of LacI**∆**1-62/DNA complex by simulated annealing method with the NMR restraint. The first 20 conformers was submitted to the PDB database [5]. The conformer 1 is the energy-lowest of 20 conformers of nonspecific complex of LacI**∆**1-62/DNA, which is the best ensemble, as stated in the PBD file (1OSL) [5]. Nearly all relative studies concerning MD simulation choose one of the 20 conformers as the initial conformation [6-8]. We have contacted with Kalodimos *et al*. who suggested using conformer 1. In this study, we choose conformer 1 as the initial conformation. (In such dynamic studies, the molecules keep shifting their conformations, so the initial conformation is not expected to have a significant effect on the result.) While in DelPhi calculation, the dynamic motion of atoms in molecules is not included. Therefore, we also showed the result from the available 20 conformers in the supporting information for a comparison (Fig S3). Fig S1 shows available ensemble of 20 LacI**∆**1-62/DNA complexes.

**
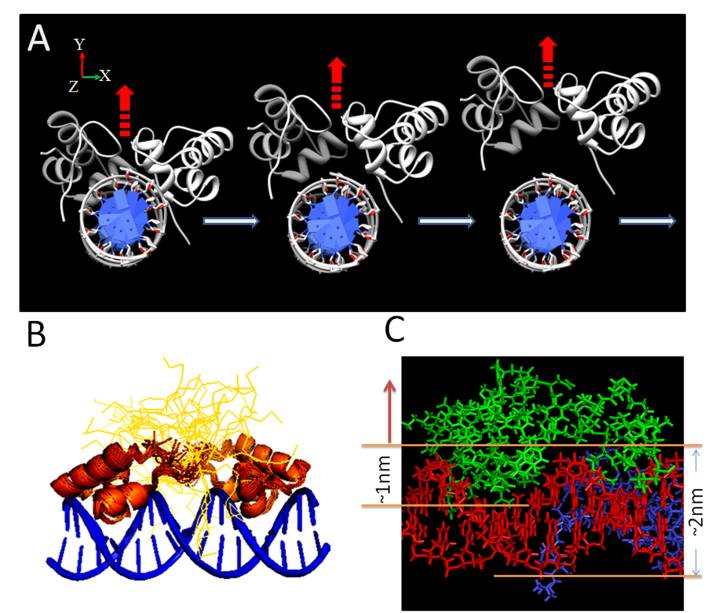
**

**Fig S1.** (A) The conformation of LacI**∆**1-62/DNA complex after rotation and the direction in which the LacI is pulled from DNA. (B) 20 conformers of the nonspecific LacI**∆**1-62/DNA complex. DNA is in blue. The DNA binding domain is in orange, and the hinge region is in yellow. (C) The molecular structure of nonspecific complex of LacI with DNA for conformer 1. The diameter of DNA (depicted in red) is ca. 2 nm, and the LacI (depicted in green and blue) is embedded ca. 1 nm in the major groove of DNA.

**Appendix D The radial distance between two monomers of the LacI and DNA during the micro-dissociation process**

We studied three kinds of systems during the micro-dissociation process: LacI**∆**1-62/DNA system, LacI**∆**1-49/DNA system and LacI**∆**1-53/DNA system. We pulled the two monomers of LacI simultaneously away from DNA along the radial direction of DNA axis (Fig S1A). The LacI complex was rotated to make the DNA axis along Z-axis. We controlled the radial distance (in Y-axis direction) between the mass center of dimer and DNA in the consecutive umbrella sampling window during the micro-dissociation process. The radial distance between the mass center of dimer and DNA increases linearly with window numbers in three systems. The radial distance between the mass center of each monomers and DNA is roughly the same as that of dimer and DNA for all these three systems. The two monomers move together with roughly the same radial distance during the micro-dissociation process even if they are not connected with S-S bond.

**
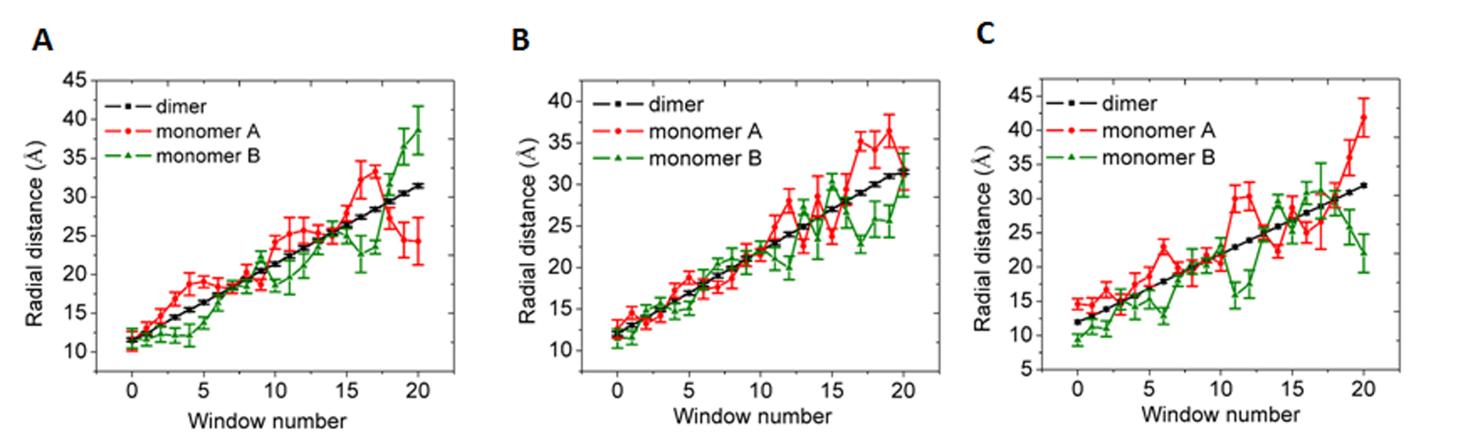
**

**Fig S2. The radial distance between two monomers of the LacI and DNA during the micro-dissociation process.** (A) The radial distance between LacI**∆**1-62 and DNA in every window. (B) The radial distance between LacI**∆**1-53 and DNA in every window. (C) The radial distance between LacI**∆**1-49 and DNA in every window

**Appendix E** **Salt dependence of the electrostatic energy of LacI/DNA with or without hinge region for 20 conformers**

In order to find the contribution of hinge region to electrostatic energy, we calculate the electrostatic energy for 20 conformers at salt concentration I = 0.001 M, 0.005 M, 0.01 M, 0.05 M and 0.1 M for 20 conformers. We found that without the hinge region, the averaged electrostatic energy of 20 conformers increases, and the averaged salt dependence of electrostatic energy becomes weaker.


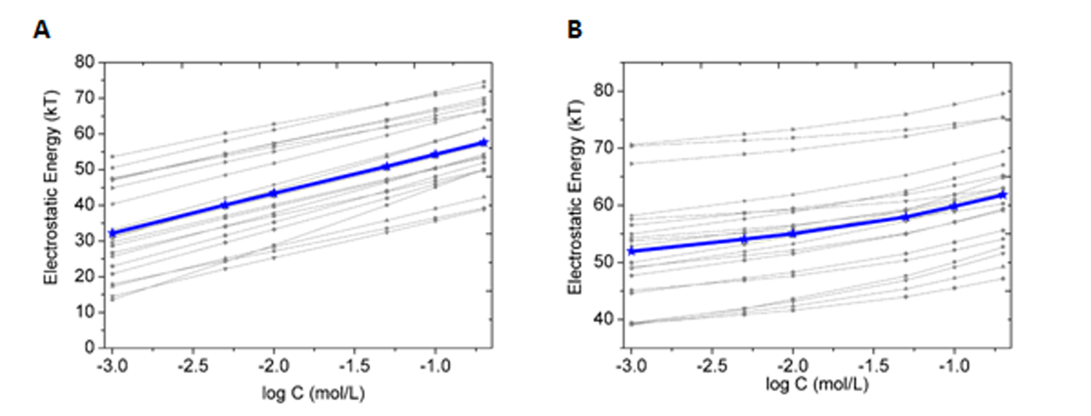


**Fig S3**. **The electrostatic energy of LacI/DNA as a function of salt concentration C.** (A) with or (B) without hinge region. The light lines are the electrostatic energy of every conformer, and the blue line is the average value for 20 conformers.

**Appendix F Characterization of the ion atmosphere of LacI/DNA based on the results of the MD simulations**

The MD trajectories obtained from the PMF calculations for LacI/DNA were analyzed to characterize the change in the ion atmosphere of DNA upon binding of the LacI. For this analysis, we computed the number of Na and Cl ions confined within distance R from the center of the helix, Figs S4A-B and the net charge of ions confined within R, Fig 4C. To access the effect of protein binding, we performed this analysis for the system where the protein and DNA formed a tight complex, and the system where the protein and DNA were separated by 20 Å of COM distance. The difference in the net charge of the ion atmosphere between the two systems (Fig S4D) indicates that the binding of LacI**∆**1-62 to DNA displaces, on average, six to eight ions from the DNA surface. For the protein variant lacking the hinge region (residue 1-49), the number of displaced ions is considerably smaller, between two and four.

**

**

**Fig S4**. **Analysis of the ion cloud based on the results of MD simulations.** (A and B) The number of Na, Cl, ions confined within distance R from the DNA’s central axis for several systems simulated using the all-atom MD method. Data for the systems where the protein and DNA are forming a tight complex are shown as black squares, (LacI**∆**1-62), red circles (LacI**∆**1-53), and blue triangle (LacI**∆**1-49). Data for the system where the DNA and the protein are separated by 20 Å of COM distance are shown as cyan triangles. (C) The net charge of ions confined within distance R from the DNA’s central axis. (D) The change in the net charge of ion cloud observed upon binding of the protein variants to DNA.

**Appendix G Analysis of electrostatic energy barrier**

Fig 4B shows an unexpected electrostatic energy barrier. It can be explained from the origin of electrostatic energy. In water solution, the electrostatic energy is composed of coulombic energy and corrected reaction field energy as shown in Eq. S2. The coulombic energy is the product of coulombic potential and the charge (Eq. S4). The calculated coulombic energy during separation process is always of negative value and increases with increasing radial distance (Fig S5A). The corrected reaction field energy is the product of polarization potential and charge (Eq. S7). The calculated corrected reaction field energy is of positive value and decreases with increasing radial distance (Fig S5A). The counterbalance between coulombic energy and corrected reaction field energy results in the positive electrostatic energy with an electrostatic energy barrier. The value of internal dielectric constant (INDI) and external dielectric constant (EXDI) affects the electrostatic energy (Fig S5B). The EXDI is usually set as the dielectric constant of water (80.00). The INDI is the dielectric constant of biomolecules (such as protein or DNA), which often ranges from 2.0 to 4.0 according to the polarizability of molecule. We used 4.0 as INDI because of numerous negative charges on DNA. When INDI=4 and EXDI=80.00, the electrostatic energy barrier is of ca. 40 kT for conformer 1 between the radial distance of 13 Å to 14 Å (Fig S5E). The electrostatic energy barrier disappears if we set INDI and EXDI at similar values (Fig S5C and S5D).


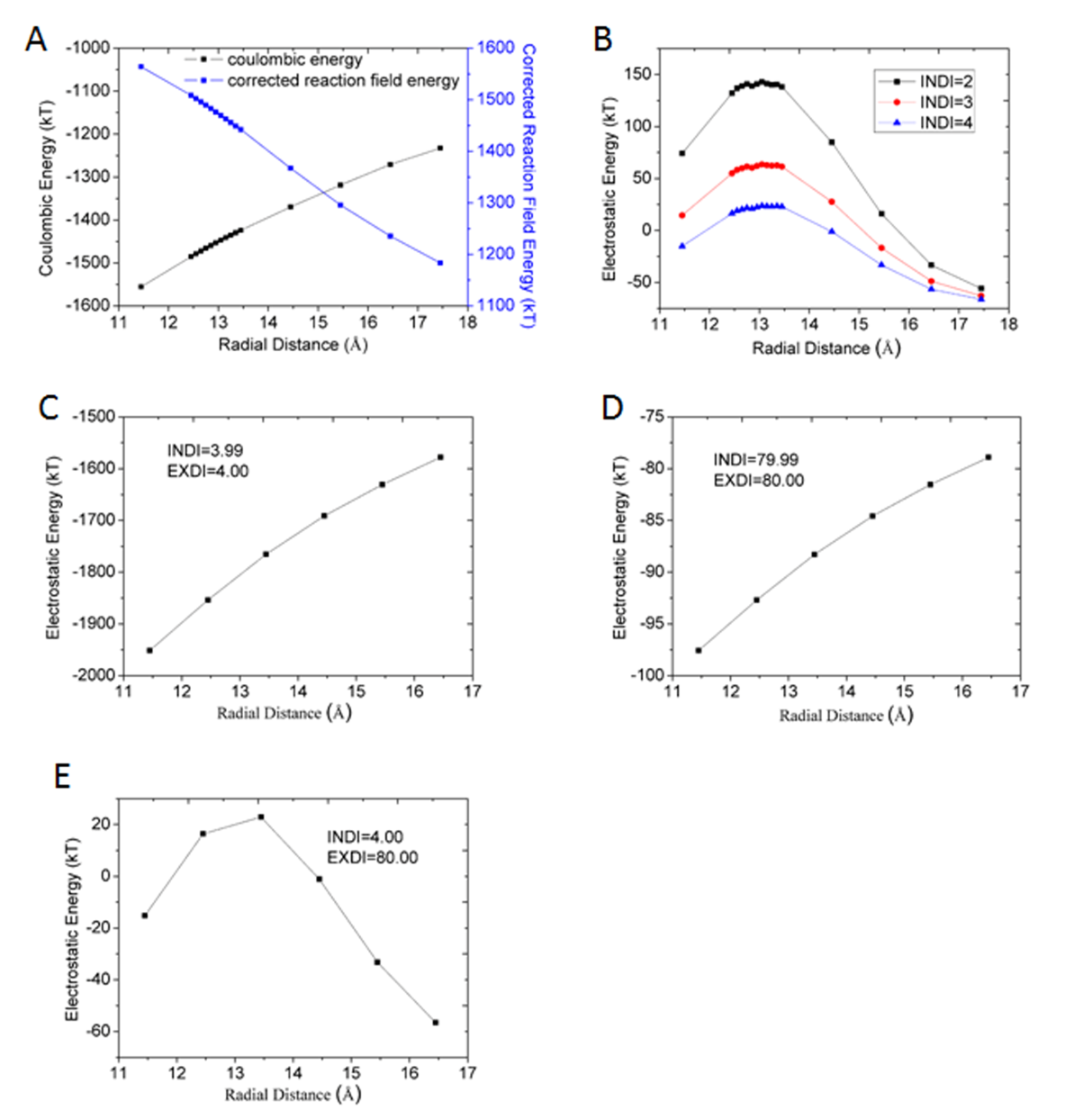


**Fig S5.** **The electrostatic energy as a function of radial distance.** (A) The coulombic energy and the corrected field energy as a function of radial distance. (B) The effect of INDI on the electrostatic energy with EXDI as 80.00. (C) The electrostatic energy as a function of radial distance with INDI=3.99 and EXDI=4.00. (D) The electrostatic energy as a function of radial distance with INDI=79.99 and EXDI=80.00. (E) The electrostatic energy as a function of radial distance with INDI=4.00 and EXDI=80.00.

**Appendix H The temperature, volume, total energy and number of water molecules in the umbrella sampling windows**

We extracted the data of temperature, volume, total energy and the number of water molecules between LacI∆1-62 and DNA. The temperature, volume, total energy and the number of water molecules keeps stable after 0.2 ns. (Fig S6 shows the first window and Fig S7 shows the last window in the umbrella sampling).


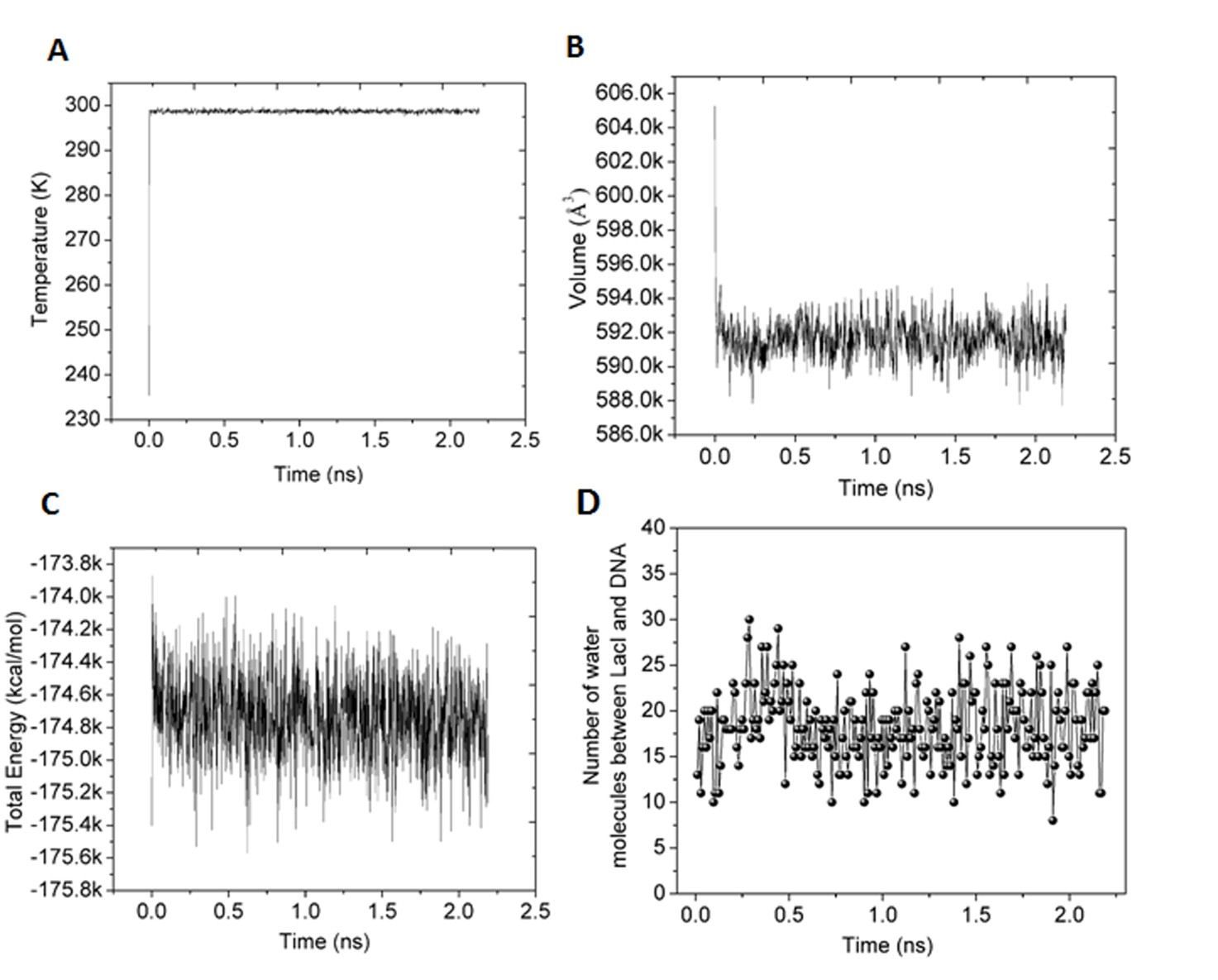


**Fig S6.** **The parameters for the LacI∆1-62/DNA system during NPT simulation.** (A) The temperature as a function of time for the first window in the umbrella sampling. (B) The volume as a function of time for the first window in the umbrella sampling. (C) The total energy as a function of time for the first window in the umbrella sampling. (D) The number of water molecules between LacI**∆**1-62 and DNA for the first window in the umbrella sampling. We defined the space between LacI**∆**1-62 and DNA using the command "within 2.5 Å of protein and within 2.5 Å of nucleic acid".


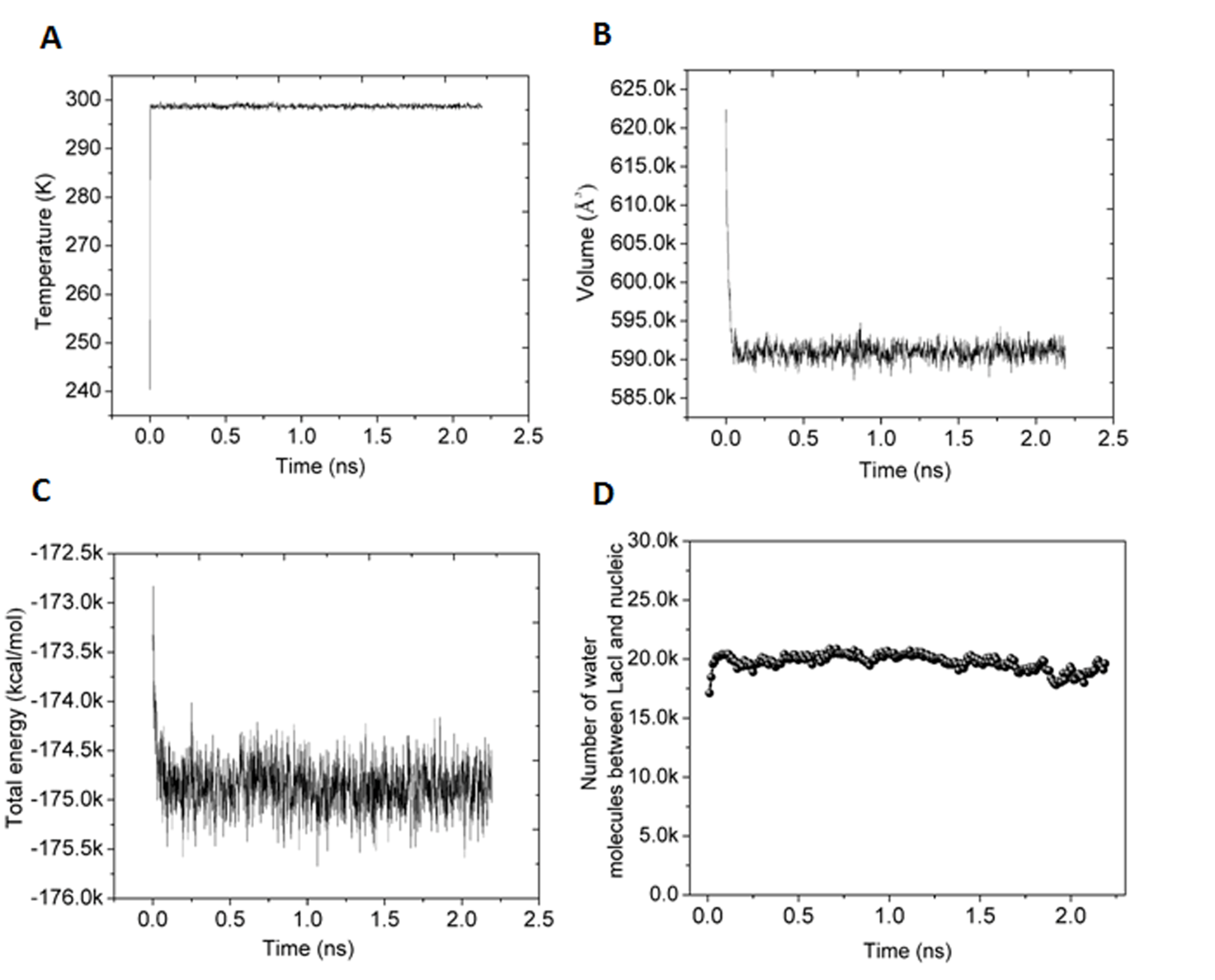


**Fig S7.** **The parameters for the LacI∆1-62/DNA system during NPT simulation.** (A) The temperature as a function of time for the last window in the umbrella sampling. (B) The volume as a function of time for the last window in the umbrella sampling. (C) The total energy as a function of time for the last window in the umbrella sampling. (D) The number of water molecules between LacI**∆**1-62 and DNA for the last window in the umbrella sampling. We defined the space between LacI**∆**1-62 and DNA using the command "within 20 Å of protein and within 20 Å of nucleic acid" in VMD.

**Appendix I The proof for the reproducibility of PMF curve for the LacI∆1-62/DNA and LacI∆1-49/DNA simulation system**

In order to prove that the PMF curve is repeatable for the LacI∆1-62/DNA and LacI∆1-49/DNA simulation system, we prolonged the simulation time twice and split the umbrella sampling simulation time into halves to calculate the PMF curves. The three PMF curves calculated from the full and each split simulation time agree well with each other. Fig S8A showed the convergence of PMF for LacI∆1-62/DNA simulation system (with hinge region). Fig S8B showed the reproducibility of PMF for LacI∆1-49/DNA simulation system (without hinge region).


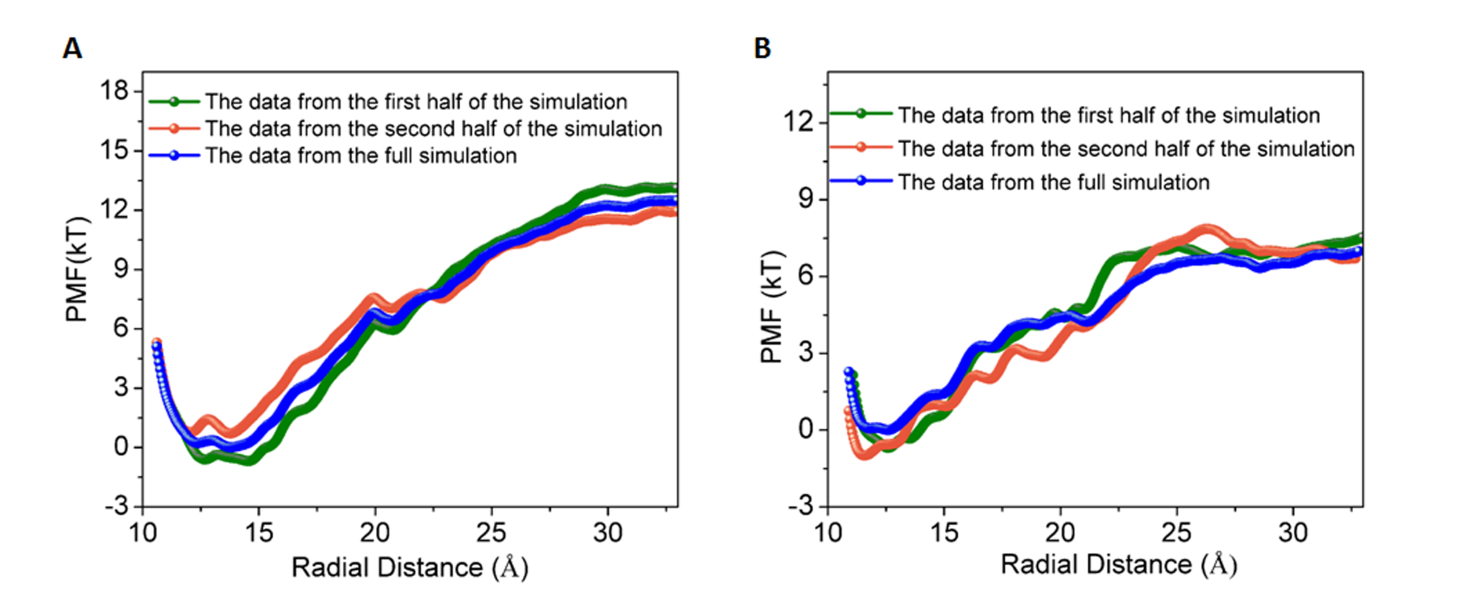


**Fig S8.** **The identification for the convergence of PMF curve for the LacI∆1-62/DNA and LacI∆1-49/DNA simulation system**. (A) The PMF curve as a function of time for the LacI∆1-62/DNA system (with hinge region). (B) The PMF curve as a function of time for the LacI∆1-49/DNA system (without hinge region).

**Appendix J The window overlap for the LacI∆1-62/DNA and LacI∆1-49/DNA simulation system**

We have identified that sufficient overlap between windows has been achieved for the LacI∆1-62/DNA and LacI∆1-49/DNA simulation system shown in Fig S9.

**
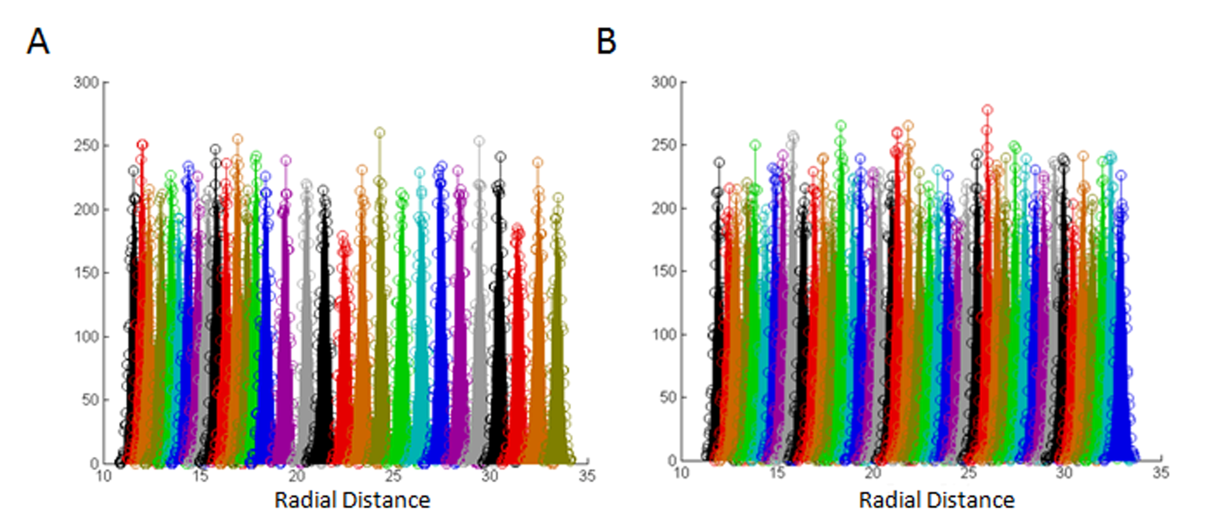
**

**Fig S9.** **The window overlap for the LacI∆1-62/DNA and LacI∆1-49/DNA simulation system.** (A) The window overlap for the LacI∆1-62/DNA system (with hinge region). (B) The window overlap for the LacI∆1-49/DNA system (without hinge region).

**Appendix K The electrostatic energy of nonspecific LacIΔ1-62/DNA and LacIΔ1-49/DNA as a function of radial distance**

We calculated the electrostatic energy of nonspecific LacIΔ1-62/DNA and LacIΔ1-49/DNA as a function of radial distance using the available 20 conformers in PDB database. We could see that without hinge region, the electrostatic energy increase. Therefore, the hinge region plays an important role in strengthen the electrostatic interaction between LacI and DNA.


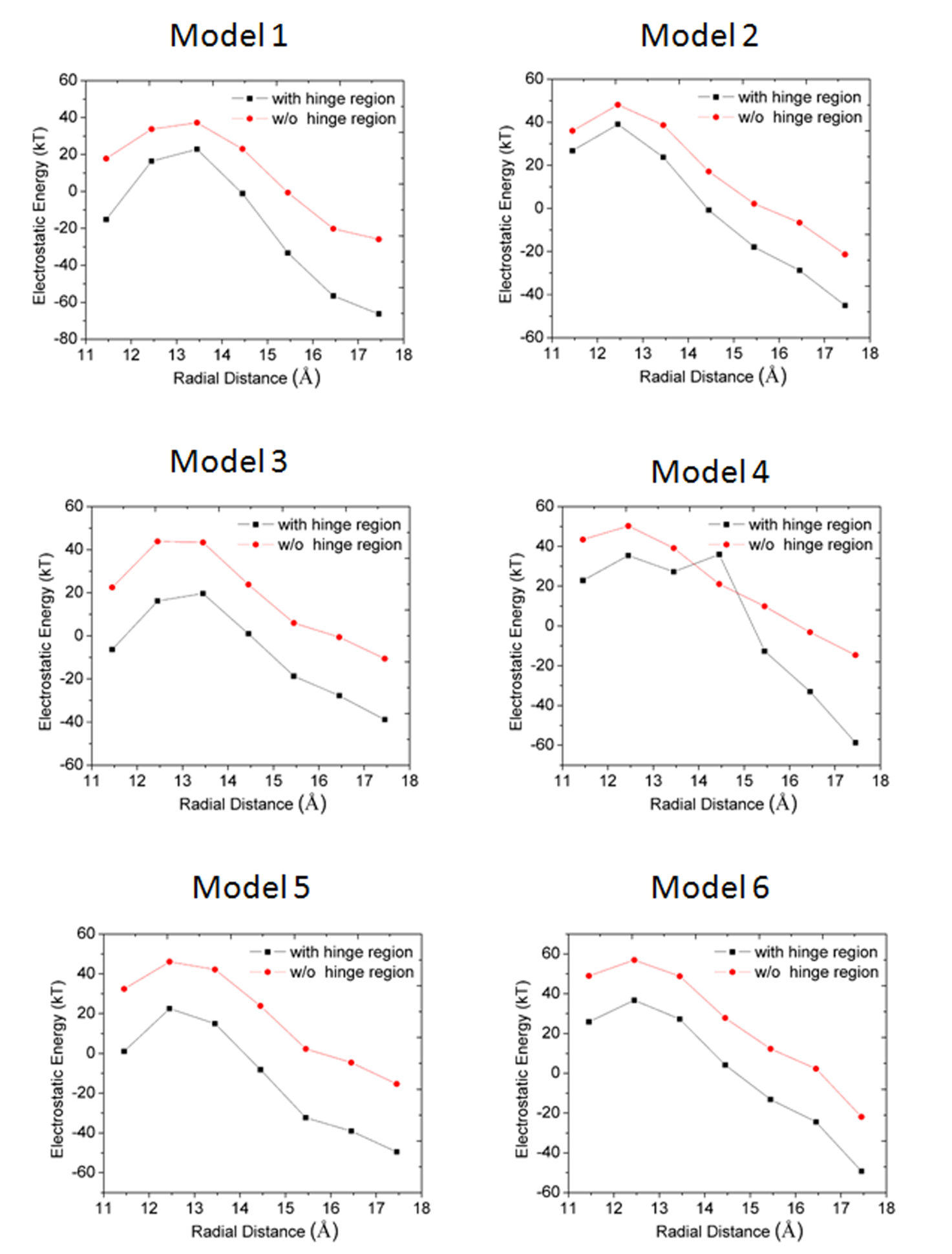


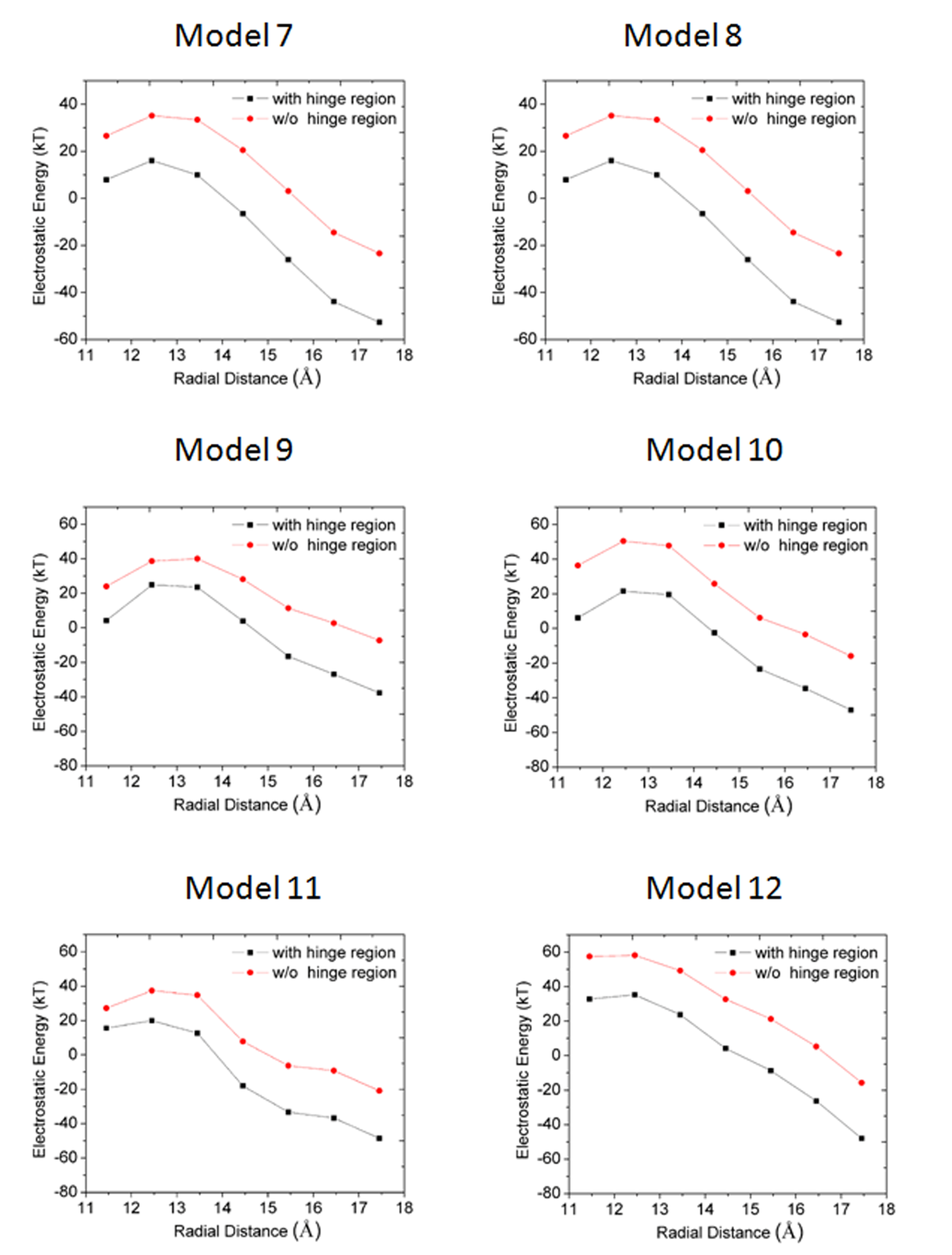


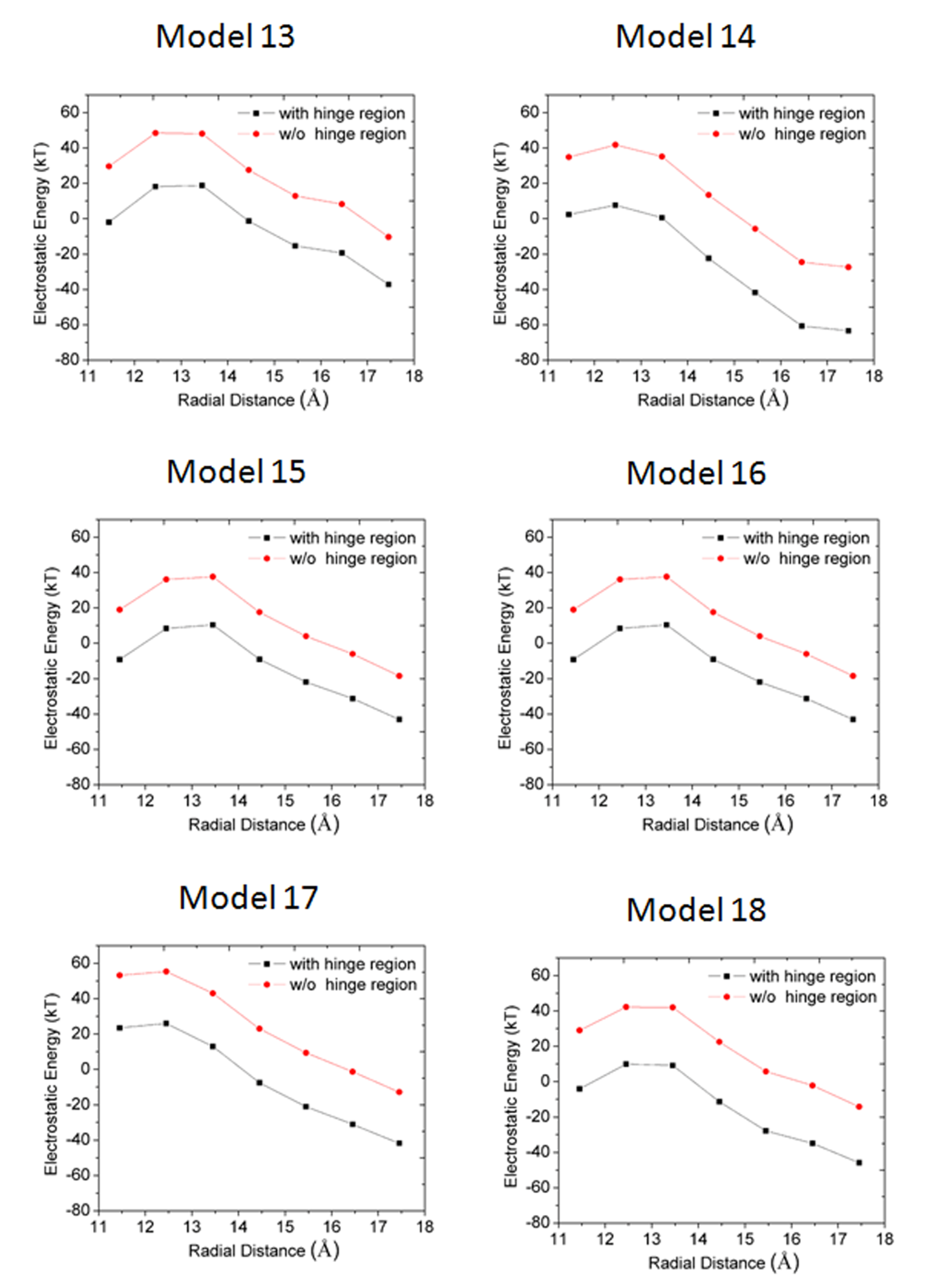


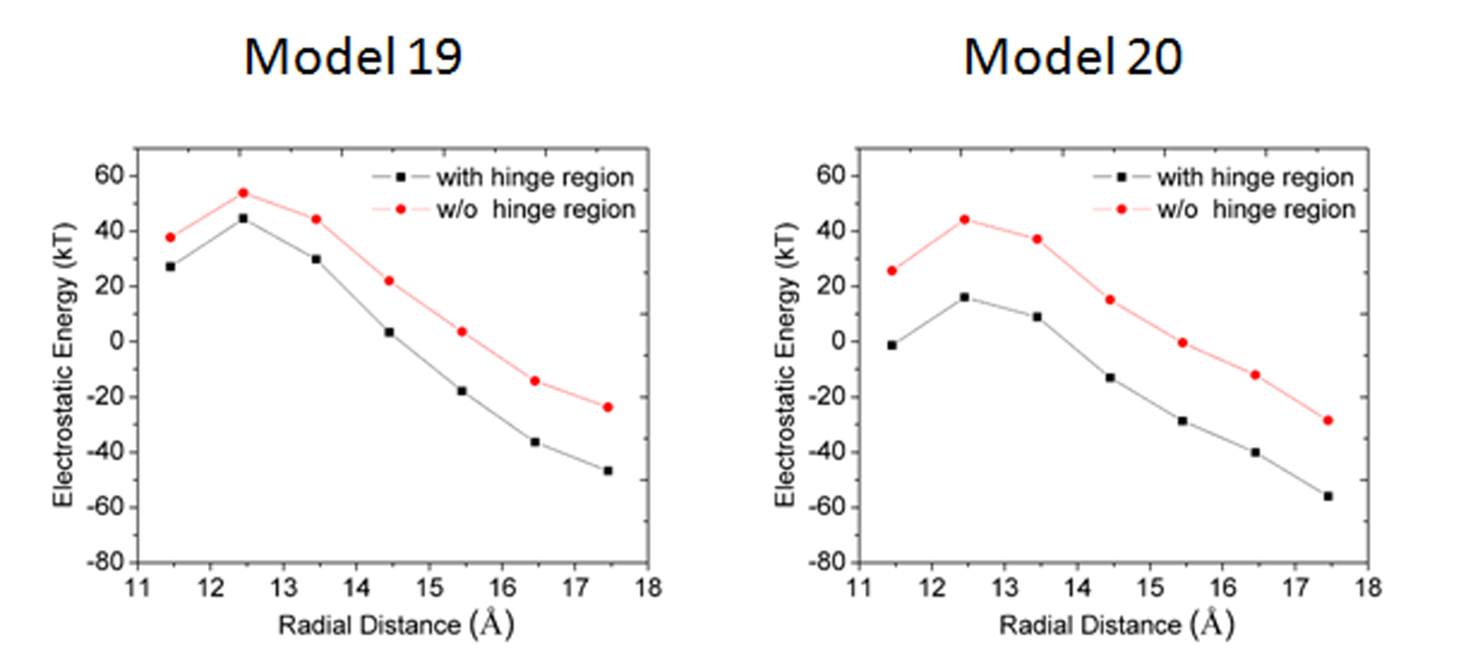


**Fig S10 The electrostatic energy of LacI-DNA as a function of radial distance for 20 conformers.**

**References:**

1. Rocchia W, Alexov E, Honig B. Extending the applicability of the nonlinear Poisson-Boltzmann equation: multiple dielectric constants and multivalent ions. J Phys Chem B. 2001;105: 6507-6514.

2. Record MT, Dehaseth PL, Lohman TM. Interpretation of monovalent and divalent-cation effects on Lac repressor-operator interaction. Biochemistry. 1977;16: 4791-4796.

3. Record MT, Lohman TM, Dehaseth P. Ion effects on ligand-nucleic acid interactions. J Mol Biol. 1976;107: 145-158.

4. Garcia-Garcia C, Draper DE. Electrostatic interactions in a peptide-RNA complex. J Mol Biol. 2003;331: 75-88.

5. Kalodimos CG, Biris N, Bonvin AMJJ, Levandoski MM, Guennuegues M, Boelens R, et al. Structure and flexibility adaptation in nonspecific and specific protein-DNA complexes. Science. 2004;305: 386-389.

6. Furini S, Barbini P, Domene C. DNA-recognition process described by MD simulations of the lactose repressor protein on a specific and a non-specific DNA sequence. Nucleic Acids Res. 2013;41: 3963-3972.

7. Furini S, Domene C, Cavalcanti S. Insights into the sliding movement of the lac repressor nonspecifically bound to DNA. J Phys Chem B. 2010;114: 2238-2245.

8. Marklund EG, Mahmutovic A, Berg OG, Hammar P, van der Spoel D, Fange D, et al. Transcription-factor binding and sliding on DNA studied using micro- and macroscopic models. P Natl Acad Sci USA. 2013;110: 19796-19801.
